# Investigating Placebos and Controls Used in Large Language Model–Based Chatbot Intervention Trials: Protocol for a Methodological Review

**DOI:** 10.2196/90507

**Published:** 2026-03-17

**Authors:** Leo Druart, Vanda Faria, Marco Annoni, John Torous, Moa Pontén, Charlotte Blease

**Affiliations:** 1 Participatory eHealth and Health Data Research Group, Department of Women’s and Children’s Health Uppsala University Uppsala, Uppsala Sweden; 2 Department of Radiology Warren Alpert Medical School Brown University Providence, RI United States; 3 Brain and Eye Pain Imaging Lab, Pain and Affective Neuroscience Center, Department of Anesthesiology, Critical Care and Pain Medicine Boston Children’s Hospital and Harvard Medical School Harvard University Boston, MA United States; 4 Comprehensive Pain Center Faculty of Medicine University Hospital Carl Gustav Carus Dresden, Saxony Germany; 5 Interdepartmental Center for Research Ethics and Integrity National Research Council Milan, Lazio Italy; 6 Department of Psychiatry Beth Israel Deaconess Medical Center, Division of Digital Psychiatry Harvard Medical School Boston, MA United States; 7 Department of Clinical Neuroscience Centre for Psychiatry Research Karolinska Institutet Stockholm, Stockholm Sweden; 8 Stockholm Health Care Services Stockholm, Stockholm Sweden

**Keywords:** large language models, chatbots, digital health, control conditions, methodological review

## Abstract

**Background:**

Large language model (LLM)–based chatbots are rapidly being repurposed as patient-facing digital health tools. Their interactive, adaptive, and seemingly empathic behavior can heighten engagement and expectancy—nonspecific factors that complicate causal inference. Yet, comparator strategies in LLM trials are inconsistently defined and often undermatched (eg, minimal education vs highly engaging chatbots), risking biased effect estimates and poor reproducibility.

**Objective:**

The aim of this study was to systematically identify and categorize the control conditions used in interventional studies of LLM-based, patient-facing digital health interventions and to evaluate their methodological appropriateness. Secondary aims are to describe variability by health domain and study design and to explore whether control type/quality relates to the direction of reported effects.

**Methods:**

This protocol follows PRISMA-P (Preferred Reporting Items for Systematic Review and Meta-Analysis Protocols) and is registered in PROSPERO. Eligible studies are interventional designs that evaluate LLM-based, patient-facing digital health interventions; any control condition is eligible (including no control, waitlist, treatment-as-usual, attention/education, active comparator, or sham digital control). We will search PubMed, PsycINFO, CENTRAL, CINAHL, and Scopus for records from January 1, 2023, onward. All records will be managed and screened in Rayyan by 2 independent reviewers. Dual, independent data extraction will target study context, intervention details, and control-arm characteristics (typology, rationale, matching to nonspecifics, blinding, reporting). No formal risk-of-bias assessments are planned, as the focus is on meta-research.

**Results:**

At submission, the protocol is registered in PROSPERO and has received no specific funding. Scoping searches are complete; full screening and extraction have not yet commenced.

**Conclusions:**

This review will provide an empirical map of control practices in LLM chatbot trials and guidance for designing better-matched comparators, supporting more valid and interpretable evaluations as LLMs diffuse into patient care.

**Trial Registration:**

PROSPERO CRD420251246148; https://www.crd.york.ac.uk/PROSPERO/view/CRD420251246148

**International Registered Report Identifier (IRRID):**

PRR1-10.2196/90507

## Introduction

### Background

Since ChatGPT’s launch in November 2022, interest in patient-facing conversational agents whose replies are generated by large language models (LLM), in other words, LLM-based chatbots, has surged, with growing attention to their potential clinical applications. Just a year after its launch, already 1 in 5 primary care physicians in the United Kingdom reported using it in their professional activities [1]. While the application of LLMs in health care presents significant opportunities to improve health care, for example, by diminishing the administrative burden for clinicians while also helping patients through its accessibility and availability, they also invite significant challenges that warrant consideration [2,3].

Now, patients are using these chatbots for their health care needs. For example, by early 2025, 10% of Australians had already used ChatGPT for health-related questions, with 61% asking questions about taking action that typically require clinical advice [4]. Of the participants who had not yet used ChatGPT for a health-related question, 40% reported that they would do so within the next 6 months. More striking, in October 2025, OpenAI announced that, every week, 1.2 million users disclosed thinking about taking their own lives with ChatGPT [5]. It is clear that these tools are being repurposed for lifestyle counselling or mental health support. Studies demonstrate that these chatbots can simulate empathy, personalize responses, and sustain meaningful dialogue, potentially amplifying expectancy and engagement effects beyond those seen in rule-based apps [6].

One key challenge, however, lies in measuring their efficacy and determining whether current methodological tools are adapted to assess these new interventions. While rigorous trials are a prerequisite for introducing new drugs to patients, LLM tools have not been subjected to similar scrutiny. Ideally, an LLM should only be used as a health intervention after safety and effectiveness have been demonstrated [7].

Such rapid dissemination raises concerns that deployment may have outpaced the development of appropriate evaluation practices. Like other nonpharmacological interventions such as surgery, physiotherapy, or psychotherapy [8], LLMs, and more generally digital interventions, pose unique challenges for evaluation when compared to drugs. Their effects often rely on multiple components, and they are, as interventions, highly adaptive. Moreover, they inherently depend on user engagement, interface design, and personalization, all of which could potentially influence outcomes independently of any hypothesized active ingredient. As the field evolves, these methodological specificities will need greater methodological attention [9]. Specifically, methodological attention should focus on designing appropriate control groups for evaluating these tools. Controls are necessary to sift out any confounding factors and accurately assess the efficacy of LLM interventions [10]. Yet, adequate controls are challenging to design and implement [11]. These considerations are especially salient for unsupervised LLMs whose conversational, adaptive, and apparently empathic responses may heighten engagement and expectancy effects [6,12,13]. In this context, it is important to distinguish between placebo responses—any observed change following a placebo intervention, and placebo effects, proper, which refer specifically to the component of that response attributable to placebo mechanisms such as expectancy or conditioning [11,14].

This is why the choice of control condition is fundamental to assess the efficacy of these tools—a consideration that has not only scientific but also ethical implications [12]. Foundational guidance for nonpharmacological trials emphasizes that controls should be selected to answer a specific scientific question while accounting for nonspecific factors such as time, attention, therapeutic alliance, and expectancy effects [15,16]. In LLM trials, an adequate control may need to match not only time and attention but also the look-and-feel and level of interactive engagement (eg, notifications, chat, interface aesthetics) to approximate a placebo control that isolates the hypothesized mechanism of action [17]. Without such methodological matching, apparent benefits may be inflated (if controls are minimally engaging) or genuine effects obscured (if controls inadvertently contain active elements), limiting the validity of the findings.

Recent scholarship has begun to map the challenges in digital health more generally. Goldberg and colleagues [17] proposed a typology of 11 control condition types used in mobile health randomized trials, along with a decision framework and a gradation of comparison strength, illuminating how different control choices afford different inferences. Methodological work has urged for comparators that better approximate what some researchers have dubbed “active placebo” characteristics in digital trials—mirroring the active arm’s interactivity and engagement while withholding the hypothesized mechanism [17,18]. A systematic review of digital shams in digital therapeutics highlighted practical and conceptual challenges—among them, inconsistently defined controls, inadequate matching on engagement, and limited reporting [18].

These contributions underscore that controls are heterogeneous, variably justified, and unevenly reported in digital intervention trials and that stronger standards are needed to ensure valid inference and comparability across studies. However, this has yet to be done for LLM-based interventions, which are increasingly gaining popularity.

Scoping reviews show that evaluation methods for LLMs are lacking in high-quality effectiveness research. The evaluation of LLMs in coaching and lifestyle has been suggested to present a lack of methodological standardization and a general trend toward less rigorous evaluation designs [19]. Similar findings were reported for LLMs used in mental health [20]. Moreover, effectiveness has been defined subjectively in over two-thirds of studies evaluating LLMs to provide health advice [21]. These findings highlight that the need for improved control designs is particularly acute for LLM, given their widespread use has advanced faster than their systematic evaluation.

In summary, the exponential growth of LLMs used as patient-facing digital health tools and the unique methodological challenges they present create a critical empirical gap: we lack a consolidated mapping of the control conditions actually used across these interventional studies of LLM-based chatbots. We also need to better understand how consistently these controls are justified and described and the extent to which they match nonspecific factors that are especially salient for conversational/artificial intelligence (AI) systems. Moreover, while guidance documents (eg, CONSORT-EHEALTH [Consolidated Standards of Reporting Trials of Electronic and Mobile Health Applications and Online Telehealth]) have improved reporting expectations, they do not extend to control adaptations such as which control to use for a given scientific aim, and evidence suggests that control selection and labeling (eg, treatment as usual, education, waitlist) remain inconsistent across the literature [17,22]. A methodological synthesis focused specifically on LLM-based interventions, where conversationality and perceived intelligence may intensify nonspecific effects, can provide the field with a clearer description of practices, highlight reporting deficits, and inform design decisions in upcoming trials.

Accordingly, this protocol describes a methodological review that aims to answer the question: what type of control conditions are used in interventional studies of LLM-based, patient-facing digital health interventions, and how methodologically appropriate are these controls?

### Objectives

This methodological review aims to systematically identify and categorize the control conditions used in interventional studies of LLM-based chatbot interventions and to evaluate their methodological appropriateness—specifically, whether a clear rationale is provided, whether placebo response components are adequately matched, and whether risks of blinding failure or contamination are addressed and reported transparently. We will also describe how control design and reporting vary across 3 health domains (mental health, chronic disease, lifestyle changes) and study designs and explore whether the type or quality of control relates to the direction of reported intervention effects (positive, null, or negative findings).

The population consists of individuals in any health domain who are intended users of an AI-based digital health tool. The intervention is an LLM-based, patient-facing digital modality, and the comparator may be any control condition, including no control, a waitlist, treatment-as-usual, attention/education, an active comparator, or another type of intervention control. The outcomes of the review include the methodological description, classification, and appraisal of control arms rather than clinical effectiveness; notwithstanding, the quality of the former will be relevant to measuring the latter.

## Methods

### Study Design

This is a protocol for a methodological (meta-research) review reported in accordance with PRISMA-P (Preferred Reporting Items for Systematic Review and Meta-Analysis Protocols) [23]. Where relevant, general methods are informed by the Cochrane Handbook [24]. Methodological reviews identify inconsistencies, gaps, and sources of bias in the design, conduct, and reporting of research. They are particularly useful when an area of research is rapidly evolving and lacks standardization, allowing researchers to map current practices and evaluate their adequacy [25]. Given the heterogeneity and emerging nature of control conditions in LLM-based digital health interventions, this approach enables a rigorous assessment of whether existing trials use appropriate comparators and adhere to methodological best practices.

### Eligibility Criteria

We will include interventional studies evaluating LLM-based health interventions, including randomized controlled trials, nonrandomized trials, quasi-experimental studies, and single-group trials (pilot/feasibility studies). Published study protocols may be considered. We will exclude observational designs (cohort, cross-sectional, case-control), case reports, editorials, reviews and commentaries, and non–peer-reviewed publications.

Regarding the population, we will include studies with individuals of any age/sex/health status who are intended users of the intervention with no restriction by clinical domain. Subpopulation analyses will be conducted for mental health, lifestyle change, or chronic disease management interventions.

The interventions concerned will be LLM-based chatbots used as patient-facing digital health interventions in which LLM constitutes the active ingredient (eg, mobile apps, web platforms, chatbots/virtual agents, generative-AI tools). We will exclude studies where the digital component serves only as a delivery vehicle (eg, videoconferencing for standard therapy) and interventions that are not directly patient-facing. An LLM will be considered the active ingredient when it generates patient-facing linguistic output intended to contribute to the intervention experience, including dialogue, explanation, summarization, or rephrasing. This reflects theoretical and empirical work suggesting that AI-generated language itself may influence expectancy, meaning-making, or engagement [11,26]. By contrast, LLM uses limited to back-end processing, or clinician-facing functions will be excluded.

Regarding controls, any control condition is eligible, including none (eg, no-treatment), waitlist, treatment-as-usual, active comparators, attention controls, placebo/sham digital controls, or standard care. Lastly, clinical outcomes will not be the eligibility criteria in this review. As a methodological review, our primary outcomes concern the characteristics of the control arms used in the included studies (see “Data items”). Included publications will be in English and published from January 2023 onward to reflect the adoption and investigation of LLMs. This cutoff was selected because it marks the point at which patient-facing, conversational LLM-based chatbots became both technically plausible and widely deployed at scale. Earlier generative or GPT-based systems were typically experimental, constrained, or not deployed as adaptive patient-facing interventions, and are therefore methodologically distinct from the intervention class examined in this review. An additional sense of urgency into the effectiveness of these tools is required because LLM-based chatbots are reported being adopted at scale by consumers and patients. For example, OpenAI reported in 2025 that 1.2 million adults every month disclosed suicidal thoughts to ChatGPT [5] and a recent UK study found that 37% of adults had used the tools for emotional or mental health support [27].

### Search Strategy

We will search 5 major bibliographic databases: PubMed, PsycINFO via EBSCO, CENTRAL via Cochrane Library, CINAHL via EBSCO, and Scopus. We will screen references of included studies and relevant reviews and consider backward and forward citation tracking. The coverage period will begin on January 1, 2023, and the search will be performed by the Uppsala University librarian team on the project (Görel Sundström and Emma Kristina Carlsson). Given the rapid pace of development in this field, we will conduct an updated search and incorporate any newly identified studies before submitting the manuscript or during the peer-review process.

The final database-specific strategies will be designed in collaboration with study librarians. The search strategies will be peer-reviewed in accordance with the PRESS 2015 framework [28]. The search strategy will be designed for PubMed using Medical Subject Headings (MeSH) and then adapted to the other databases and their thesauri. Terms will also be searched for in the title/abstract of publications. The PubMed search string is available in Multimedia Appendix 1.

### Data Management and Selection Process

Records will be managed in Rayyan for deduplication and screening [29]. Screening will proceed in two stages, that is, (1) titles/abstracts and (2) full texts, conducted independently by two reviewers through the Rayyan software. Disagreements will be resolved by discussion or by a third reviewer. We will report a PRISMA 2020 flow diagram for identification, screening, eligibility, and inclusion [30]. Prior to formal screening, we will pilot test on a smaller sample of studies. Any disagreements at this stage will be discussed and help refine the screening process to ensure consistent interpretation.

### Data Extraction

At least two reviewers will independently extract data by using an extraction form. Disagreements will be resolved through discussion or by a third reviewer. Where intervention or control details are unclear, we will attempt to contact the author (via one initial email and one reminder). We will record when information is not reported or unclear. Extracted data are presented in Table 1.

**Table 1:** Planned extracted data.

Type of data	Data
Study identification	First author name Year of publication Country of study
Study design and sample	Study design (options: randomized controlled trial, nonrandomized trial, single group) Sample size Participant characteristics (age, gender, clinical condition, self-report or clinical diagnosis, verification of self-reported data) Recruitment (online, in-person) Follow-up characteristics (length of study, compensation or incentives) Research aim (copy research aim) Requires a control group? (deduced from research aim, possible options: yes, no, unsure)
Intervention characteristics	Name/description of large language model intervention Mode of delivery (eg, mobile app, web-based content) Target condition or domain Duration, frequency, and intensity of the intervention Supposed mechanism of action explained (yes/no) Supposed mechanism of action (detail in free text)
Comparator characteristics	Type of control condition Type of control condition (describe in free text) Justification for choice of control explained (yes/no) Justification for choice of control (detail in free text) Blinding status (participants, providers, assessors, analysts)
Methodological appropriateness	Given the study design, research aim, need for control, intervention description, supposed mechanism of action and type of control condition, is the control group appropriate? (Yes/No) Comment on the previous answer Are placebo responses controlled for adequately? Are placebo effects controlled for adequately?
Outcomes and results	Conclusion on primary outcome (positive finding, null finding, unclear)

### Outcomes

The primary outcome of this review is the type and frequency of control conditions used. The absence of a comparator will be coded as a distinct control category (no control). Control-arm appropriateness will be evaluated relative to the stated research aim; where a comparator is required to support causal inference but absent, the study will be classified as not appropriately controlled.

Secondary outcomes will include the adequacy/appropriateness of controls with respect to the placebo response and theoretical justification; the reporting quality of control descriptions and justifications. Control-arm appropriateness will be evaluated using a predefined set of extraction items (Table 1: items in methodological appropriateness), which are considered jointly as a qualitative decision framework. These items assess whether a control condition is present, justified, aligned with the study aim, and designed to address relevant placebo response components. Appropriateness will be classified categorically rather than scored numerically, as different control choices afford different inferential aims rather than representing a linear quality continuum. Where judgments require interpretation, disagreements will be resolved through collegial discussion among authors with complementary expertise in placebo research, clinical trial methodology, and digital health interventions [11,31]. For Table 1 items regarding the methodological appropriateness of accounting for placebo responses and effects, we will consider that placebo responses refer to observed changes following intervention exposure, whereas placebo effects proper denote changes attributable to mechanisms such as expectancy or conditioning [11,14].

An exploratory outcome is to assess whether the type and quality of the control condition are associated with differences in the reported effectiveness of digital interventions, by comparing success proportions (see Table 1) across control categories.

### Risk of Bias in Individual Studies

We will not conduct a formal risk-of-bias assessment because the review does not synthesize treatment effects; instead, it characterizes control-arm design and reporting (ie, a meta-research focus). We will, however, extract bias-relevant features (eg, blinding, contamination, missing data statements) to inform the appraisal of control adequacy.

Assessment of publication bias or selective reporting at the meta-analytic level is not planned, as we do not pool effect sizes. GRADE (Grading of Recommendations Assessment, Development and Evaluation) is not applicable because we are not grading the certainty of an effect estimate [32].

### Data Synthesis

We will use descriptive statistics (counts, proportions, medians/IQRs where relevant) to summarize control-type frequencies, presence/absence of justifications, and reporting completeness. Stratified summaries will be presented by health domain (mental health, lifestyle changes, chronic disease management). For studies with more than two arms, we will treat each LLM-vs-control comparison in a multi-arm study as a separate comparison unit for exploratory analyses of outcome direction by control type.

### Amendments

Any protocol amendments (eg, eligibility refinements, added subgroup) will be documented, dated, and justified within the PROSPERO record and reported in the final manuscript [23].

## Results

**Figure 1:**
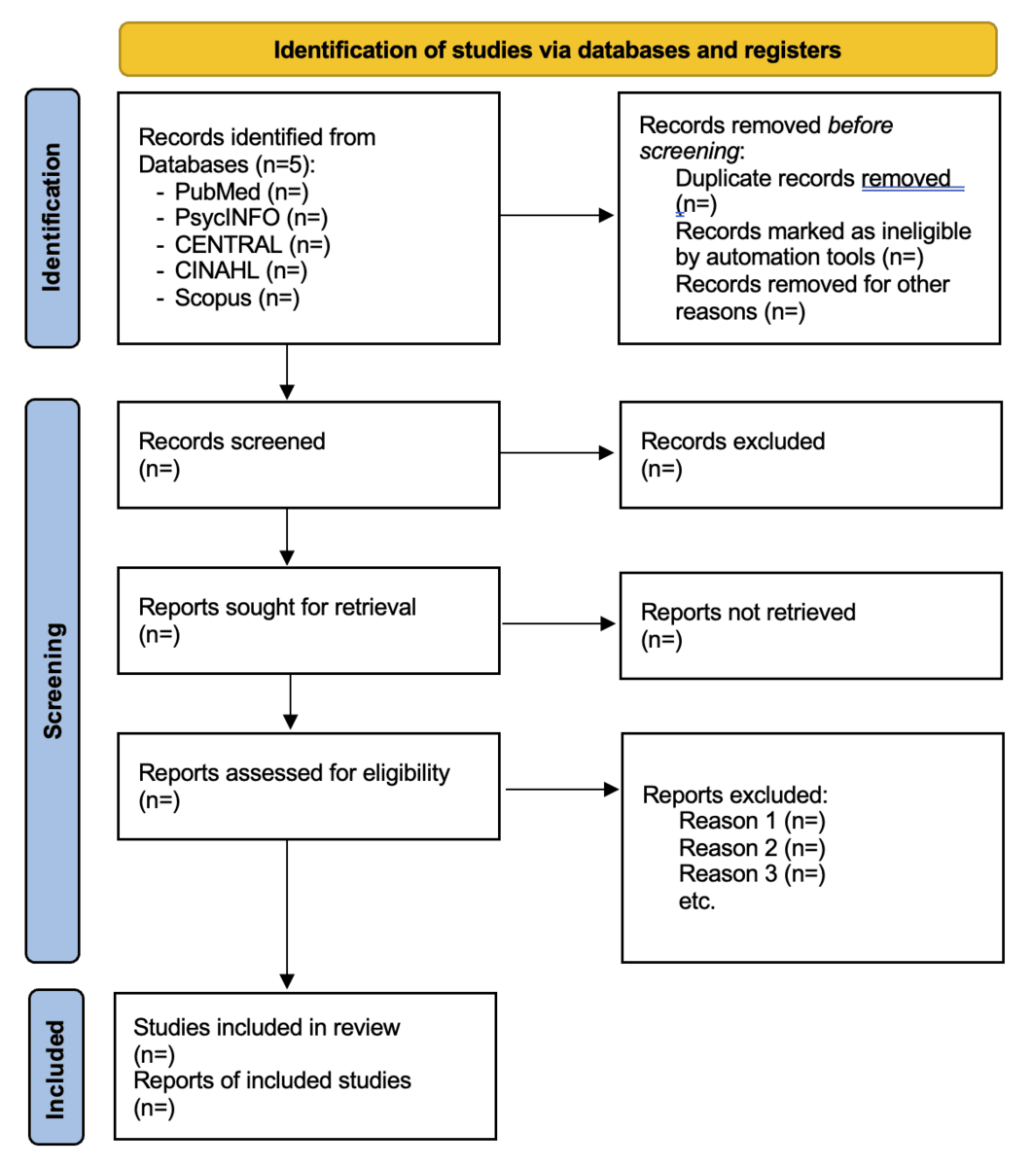
Anticipated PRISMA (Preferred Reporting Items for Systematic Review and Meta-Analysis) 2020 flow diagram for the planned study selection process.

As of submission, the protocol has been registered in PROSPERO (CRD420251246148). The database-specific search strategies have been pretested, and preliminary scoping searches have been conducted to estimate the volume and refine the eligibility criteria. No study data have been synthesized yet; this manuscript details the a priori methods that will guide the review as shown in Figure 1.

## Discussion

### Principal Results

This review will provide researchers, clinicians, and policymakers with a systematic assessment of the control conditions used to measure the efficacy of LLM-based interventions and their adequacy. This will enable a more comprehensive evaluation of the current evidence regarding the effectiveness of LLM interventions, allowing health care providers, policymakers, and, most importantly, patients to make informed decisions when considering the use of LLMs in health care.

We hypothesize that many LLM-based health trials employ control conditions that are variably defined, underjustified, and insufficiently matched for nonspecific effects, reflecting the specific challenges that evaluating digital interventions pose, yet potentially biasing inferences about the efficacy of the intervention. The outcome of this research will be highly informative for all stakeholders and will allow researchers to design more optimal control conditions in the future.

### Limitations

A key limitation of this review is that the evidence base for LLM-based digital health interventions is rapidly evolving, which restricts the strength of any conclusions about optimal control conditions. This challenge is further accentuated by the inherent delay between the emergence of new technologies, the conduct of research, and the publication of findings. Nonetheless, adoption of these systems is already occurring at scale, and the ethical risks associated with inadequate or poorly designed controls are substantial. In this context, we argue that providing an early methodological assessment is preferable to delaying synthesis until the field matures, as timely guidance may help prevent avoidable methodological weaknesses from becoming entrenched in future research.

### Comparison With Prior Work

There has yet to be any systematic mapping of the design and use of control conditions in LLM trials. However, research has been performed for digital interventions more broadly, allowing researchers to adapt reporting guidelines to the specifics of eHealth interventions through the CONSORT-EHEALTH, emphasizing the need to specifically describe components such as usability, dose/exposure, and usage metrics [22].

A previous review provided an initial examination of how control conditions were used in digital therapeutics; however, this work preceded the advent of LLMs in health care [18]. Goldberg and colleagues [17] offered a precise mapping of the typology of control conditions that were used in mobile health interventions, showing a clear need for improvement.

### Conclusions

This protocol outlines a timely and rigorous plan for a methodological synthesis of control conditions used in LLM-based health trials. By systematically classifying control arms, assessing their appropriateness, and evaluating the quality of reporting, the review will provide an empirical foundation for improved comparator design in future LLM health research. The findings are expected to inform investigators and reviewers in the rapidly evolving field of LLM-based chatbots and generative AI, where standards for comparators are still being developed.

## Appendix

Multimedia Appendix 1 PubMed search strategy.

## Abbreviations

AI: artificial intelligence
CONSORT-EHEALTH: Consolidated Standards of Reporting Trials of Electronic and Mobile Health Applications and Online Telehealth
GRADE: Grading of Recommendations Assessment, Development and Evaluation
LLM: large language model
MeSH: Medical Subject Headings
PRISMA: Preferred Reporting Items for Systematic Review and Meta-Analysis
PRISMA-P: Preferred Reporting Items for Systematic Review and Meta-Analysis Protocols

## Data Availability

This article is a research protocol and does not include any data. Data resulting from this research will be made available upon publication of the results.
