# Supplementary material for: Investigating Placebos and Controls Used in Large Language Model–Based Chatbot Intervention Trials: Protocol for a Methodological Review
Source: JMIR Res Protoc. 2026 Mar 17;15:e90507. doi: 10.2196/90507 (PMC13040165; doi:10.2196/90507)
Supplement: Multimedia Appendix 1 [file resprot_v15i1e90507_app1.docx]

Supplementary Materials

PubMED Research String :`

((("Generative Artificial Intelligence"[MeSH Terms] OR ("chat bot*"[Title/Abstract] OR "chatbot*"[Title/Abstract] OR "conversational agent*"[Title/Abstract] OR "dialog* system*"[Title/Abstract] OR "gAI"[Title/Abstract] OR "genAI"[Title/Abstract] OR "Generative Artificial Intelligence"[Title/Abstract] OR "Generative Pre-Trained Transformer"[Title/Abstract] OR "GPT"[Title/Abstract] OR "large language model*"[Title/Abstract] OR "LLM"[Title/Abstract] OR "LLM based"[Title/Abstract] OR "LLMs"[Title/Abstract] OR "virtual agent*"[Title/Abstract] OR "Anthropic"[Title/Abstract] OR "Bard"[Title/Abstract] OR "bing chat"[Title/Abstract] OR "chat gpt*"[Title/Abstract] OR "chatgpt*"[Title/Abstract] OR "Claude"[Title/Abstract] OR "Copilot"[Title/Abstract] OR "DeepSeek"[Title/Abstract] OR "Gemini"[Title/Abstract] OR "Grok"[Title/Abstract] OR "LLaMA"[Title/Abstract] OR "Le Chat"[Title/Abstract] OR "Meta AI"[Title/Abstract] OR "Mistral"[Title/Abstract] OR "OpenAI"[Title/Abstract]) OR (("Mobile Applications"[MeSH Terms] OR "Smartphone"[MeSH Terms] OR ("app"[Title/Abstract] OR "apps"[Title/Abstract] OR "cell* phone*"[Title/Abstract] OR "mobile application*"[Title/Abstract] OR "mobile phone*"[Title/Abstract] OR "smart phone*"[Title/Abstract] OR "smartphone*"[Title/Abstract])) AND ("Natural Language Processing"[MeSH Terms] OR "Machine Learning"[MeSH Terms] OR ("AI"[Title/Abstract] OR "AI based"[Title/Abstract] OR "AI driven"[Title/Abstract] OR "artificial intelligence"[Title/Abstract] OR "Machine Learning"[Title/Abstract] OR "natural language processing*"[Title/Abstract])))) AND ("Randomized Controlled Trial"[Publication Type] OR "Controlled Clinical Trial"[Publication Type] OR "Feasibility Studies"[MeSH Terms] OR "Pilot Projects"[MeSH Terms] OR ("RCT"[Title/Abstract] OR "blind stud*"[Title/Abstract] OR "blind trial"[Title/Abstract] OR "clinical trial"[Title/Abstract] OR "controlled stud*"[Title/Abstract] OR "controlled trial"[Title/Abstract] OR "doubleblind stud*"[Title/Abstract] OR "doubleblind trial"[Title/Abstract] OR "feasibility stud*"[Title/Abstract] OR "intervention* stud*"[Title/Abstract] OR "non random*"[Title/Abstract] OR "nonrandom*"[Title/Abstract] OR "pilot stud*"[Title/Abstract] OR "placebo"[Title/Abstract] OR "quasi experimental*"[Title/Abstract] OR "random*"[Title/Abstract] OR "single group trial*"[Title/Abstract]))) NOT ("Systematic Review"[Publication Type] OR "Meta-Analysis"[Publication Type] OR "Systematic Review"[Title] OR "Meta-Analysis"[Title])) AND 2023/01/01:2026/12/31[Date - Publication]
